# Reactivity of Amine/E(C_6_F_5_)_3_ (E = B, Al) Lewis Pairs toward Linear and Cyclic Acrylic Monomers: Hydrogenation *vs.* Polymerization

**DOI:** 10.3390/molecules20069575

**Published:** 2015-05-26

**Authors:** Jiawei Chen, Eugene X.-Y. Chen

**Affiliations:** Department of Chemistry, Colorado State University, Fort Collins, CO 80523-1872, USA

**Keywords:** frustrated Lewis pair, Lewis pair polymerization, amine, alane, borane

## Abstract

This work reveals the contrasting reactivity of amine/E(C_6_F_5_)_3_ (E = B, Al) Lewis pairs toward linear and cyclic acrylic monomers, methyl methacrylate (MMA) and biorenewable γ-methyl-α-methylene-γ-butyrolactone (_γ_MMBL). While mixing of 2,2,6,6-tetramethylpiperidine (TMP) and B(C_6_F_5_)_3_ leads to a frustrated Lewis pair (FLP), Et_3_N reacts with B(C_6_F_5_)_3_ to form disproportionation products, ammonium hydridoborate ionic pair and iminium zwitterion. On the other hand, the stoichiometric reaction of either TMP or Et_3_N with Al(C_6_F_5_)_3_ leads to clean formation of a classic Lewis adduct (CLA). Neither TMP nor Et_3_N, when paired with E(C_6_F_5_)_3_, polymerizes MMA, but the Et_3_N/2B(C_6_F_5_)_3_ pair promotes transfer hydrogenation of MMA to form methyl isobutyrate. In contrast, the amine/E(C_6_F_5_)_3_ pairs promote rapid polymerization of _γ_MMBL carrying the more reactive exocyclic methylene moiety, achieving full conversion in less than 3 min even at a low catalyst loading of 0.0625 mol %. TMP is more effective than Et_3_N for the polymerization when paired with either the borane or the alane, while the alane exhibits higher polymerization activity than the borane when paired with Et_3_N. Overall, the TMP/Al(C_6_F_5_)_3_ system exhibits the highest polymerization activity, achieving a maximum turn-over frequency of 96,000 h^−1^ at 0.125 mol % of catalyst loading, producing high molecular weight P_γ_MMBL with *M*_n_ = 1.29 × 10^5^ g∙mol^−1^.

## 1. Introduction

The combination of various highly acidic Lewis acids (LAs) and sterically hindered Lewis bases (LBs) provides opportunities to construct different types of “frustrated Lewis pairs” (FLPs) [1,2,3,4,5,6,7,8,9], in which the direct LA-LB interaction is either precluded or significantly weakened due to the steric crowdedness. More significantly, this unquenched, orthogonal reactivity allows the cooperative activation of small molecules [10,11,12,13,14,15,16,17,18,19,20,21,22,23,24,25], catalytic hydrogenation [26,27,28,29,30,31,32] and new reactivity/reaction development [33,34,35,36,37,38,39,40,41,42,43,44,45]. While the original FLP systems pioneered by Stephan and Erker focused mostly on boron-based LAs and phosphine-based LBs, the scopes of the suitable candidates for the FLP chemistry have recently been extended to other group 13 and 14 elements as LAs (e.g., organoaluminum species, silylium cations, and borenium cations), and group 14 and 15 elements as LBs (e.g., amines, pyridines, and carbenes). Furthermore, a considerable amount of efforts has been directed to the design of unimolecular FLPs and chiral versions of FLPs for asymmetric transformations [46,47,48,49,50,51,52,53,54,55].

**Scheme 1:**
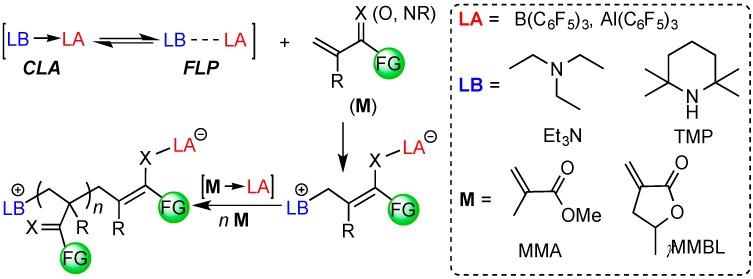
Generalized chain initiation and propagation mechanism for polymerization of conjugated polar alkenes carrying a functional group (FG) by Lewis pairs through zwitterionic active species or intermediates [56], and the structure of LAs, LBs and monomers examined in this study.

We have been interested in developing new polymerization methods enabled by Lewis pairs, namely Lewis Pair Polymerization (LPP) [56], to synthesize different classes of polymers. In this type of polymerization, Lewis pairs are involved in monomer substrate activation, chain initiation, as well as chain propagation and termination/transfer steps (Scheme 1). We have demonstrated, for example, FLPs or classic Lewis adducts (CLAs) consisting of bulky phosphine or *N*-heterocyclic carbene (NHC) LBs and the highly acidic Al(C_6_F_5_)_3_ LA are capable of promoting rapid polymerization of conjugated polar alkenes such as linear acrylic monomer methyl methacrylate (MMA) and cyclic biorenewable γ-methyl-α-methylene-γ-butyrolactone (_γ_MMBL) into high molecular weight polymers [57,58,59]. Such LPP system is also effective for the polymerization of monomers featuring the C=C-C=N functionality, including 2-vinyl pyridine and 2-isopropenyl-2-oxazoline [60,61]. Controlled Ring-opening (co)polymerization of other monomers such as lactide and lactones into cyclic polymers has been achieved by employing Zn(C_6_F_5_)_2_-based Lewis pairs [62], while radical polymerization of styrene is successfully mediated by a persistent FLP-NO aminoxyl radical generated by *N*,*N*-cycloaddition of a cyclohexylene-linked intramolecular P/B system to nitric oxide [63]. More recently, we found that the boron-based LA, in combination with various NHC or phosphine LBs, can be highly effective for polymerization of MMA and _γ_MMBL: while the B/P FLP systems exhibit no or negligible polymerization reactivity, the P→B and NHC→B CLAs have been found to exhibit unexpectedly high activity for the polymerization of _γ_MMBL [64]. This unique polymerization method has now been employed to the regio-controlled polymerization of dissymmetric divinyl polar monomers [57,65].

A search of the current array of LPP systems reveals that the combination of LAs and amines is scarce and not well-established [58]. In contrast, the B/N system has been studied extensively in the area of the FLP chemistry with respect to the LA-LB interaction, small molecule activation [25], catalysis [28,44,66,67] as well as mechanistic investigations [68]. In this context, we set out to examine the polymerization reactivity of two widely used amines, triethylamine (Et_3_N) and 2,2,6,6-tetramethylpiperidine (TMP), coupled with two strong organo-LAs, E(C_6_F_5_)_3_ (E = B, Al). We envisioned that the introduction of such sterically encumbered amines with different structural and electronic properties will present an opportunity to probe the polymerization activity of the amine/E(C_6_F_5_)_3_ (E = B or Al) system toward two representative acrylic monomers, linear MMA and cyclic _γ_MMBL (Scheme 1).

## 2. Results and Discussion

### 2.1. Reaction of Amines and E(C_6_F_5_)_3_ in the Absence of Monomer

A survey of the recent literatures indicates that the coordination chemistry and structural characterizations of several amine/E(C_6_F_5_)_3_ pairs have been reported. Preceding the discovery of the FLP system, earlier studies focused on systems featuring direct B-N and Al-N bond formation with less sterically hindered amines. For instance, Lancaster and co-workers [69] reported that both the borane and alane E(C_6_F_5_)_3_ form stable adducts with a variety of primary and secondary amines. In addition, in all of the cases, the two Lewis acids show invariable reactivity towards these amines. On the other hand, we observed markedly different polymerization activity between the borane and the alane, when used as (co)catalysts and monomer activators for both transition-metal-catalyzed and LPP processes [59,70,71,72,73]. In general, the alane is more effective than borane due to its higher Lewis acidity, oxophilicity and larger radius. Our recent work showed that the alane displays clear advantages over the borane in several different types of FLP-type catalysis [74]. In this LPP work, we chose two sterically congested amines, Et_3_N and TMP, as the LB to pair with E(C_6_F_5_)_3_ (Scheme 1).

**Scheme 2:**
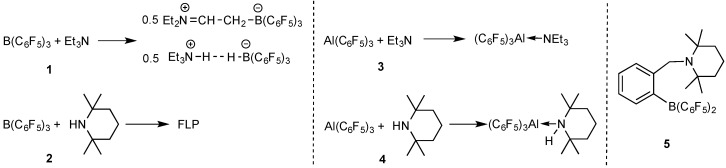
Different amine/E(C_6_F_5_)_3_ (E = B or Al) systems employed for this LPP study and their corresponding reactivity in the absence of monomer.

**Figure 1:**
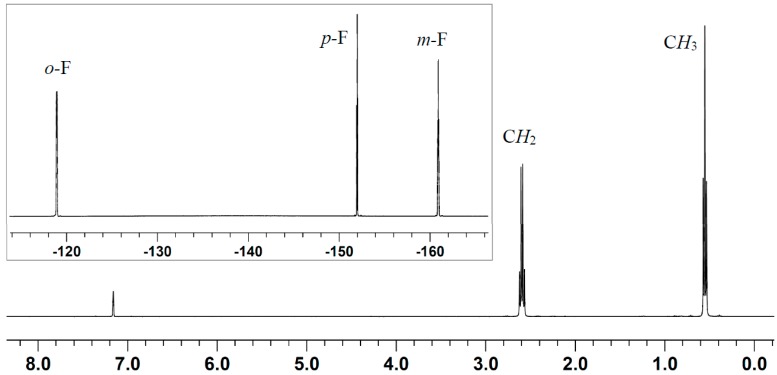
^1^H- and ^19^F- (inset) spectra (C_6_D_6_) of adduct Et_3_N·Al(C_6_F_5_)_3_.

**Figure 2:**
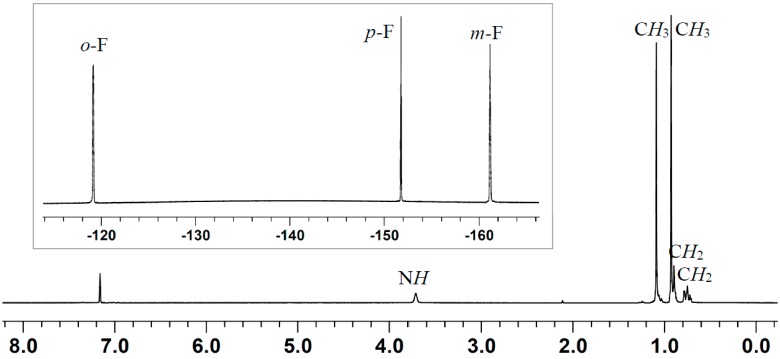
^1^H- and ^19^F- (inset) spectra (C_6_D_6_) of adduct TMP·Al(C_6_F_5_)_3_.

These two amines exhibit FLP-type reactivity when coupled with the borane [25,75], but their reaction with the alane has not yet been examined. An earlier study showed that, mixing of B(C_6_F_5_)_3_ and Et_3_N results in a disproportionation reaction of the Lewis pair to form ammonium hydridoborate (C_6_F_5_)_3_B-H···H-N(C_2_H_5_)_3_, concomitant with equimolar iminium zwitterion (C_6_F_5_)_3_B-CH_2_-CH=N(C_2_H_5_)_2_ [75] (Scheme 2). B(C_6_F_5_)_3_ is known to abstract a hydride from the α-position of a bulky amine with α-hydrogens and form an FLP with the one without α-hydrogens such as TMP. The latter system was utilized for dihydrogen activation and CO_2_ reduction [25,44]. We speculated that switching from B(C_6_F_5_)_3_ to its congener Al(C_6_F_5_)_3_ could achieve different coordination reactivity. As expected, the alane, considered possessing higher Lewis acidity and less steric hindrance, forms a dative bond with both Et_3_N and TMP. In the stoichiometric reaction between Al(C_6_F_5_)_3_ and Et_3_N, the crystalline adduct Et_3_N·Al(C_6_F_5_)_3_ was isolated in a yield of 81%. Only one set of ethyl and one set of C_6_F_5_ resonances were presented in the ^1^H- and ^19^F-NMR spectra, respectively (Figure 1). Apparently, the direct Al-N bonding prevents other reactions such as hydride abstraction, which stands in contrast to the borane. Similarly, the TMP·Al(C_6_F_5_)_3_ adduct was isolated as colorless crystals in a yield of 85%, in which the complex formation was confirmed by both ^1^H- and ^19^F-NMR spectroscopy (Figure 2). In the ^1^H-NMR spectrum, the N-*H* signal is significantly downfield shifted to 3.71 ppm (*cf.* 0.64 ppm for the free TMP), as a result of the enhanced acidity after coordination to the electron-deficient alane. This adduct is stable in solution up to 2 days, which precludes the possibility of catalyst decomposition (e.g., protonolytic cleavage of the Al-C bond by the N-H proton) during the course of polymerization (*vide infra*). In addition, an intramolecular FLP (**5**) [67] was also included for the LPP study.

### 2.2. Stoichiometric Hydrogenation of MMA to Methyl Isobutyrate *by Et_3_N/B(C_6_F_5_)_3_*

**Figure 3:**
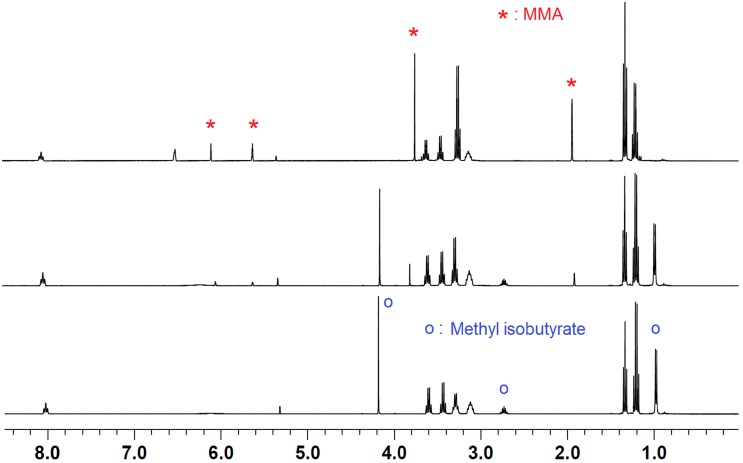
^1^H-NMR spectra (CD_2_Cl_2_) of a mixture of Et_3_N/B(C_6_F_5_)_3_/MMA in the ratio of 1:1:1 (**top**), 1:1.5:1 (**middle**) and 1:2:1 (**bottom**, some amount of Et_3_N-H···H-B(C_6_F_5_)_3_ remains due to a slight deficiency of MMA).

**Scheme 3:**
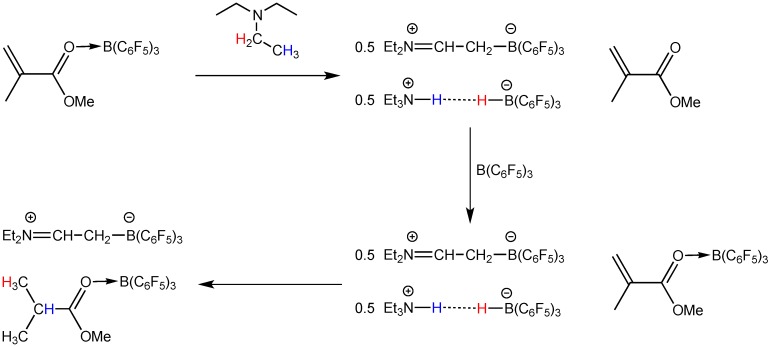
Stoichiometric hydrogenation of MMA to methyl isobutyrate by Et_3_N/B(C_6_F_5_)_3_.

We first examined the activity of the current amine/LA pairs toward polymerization of the linear methacrylate, MMA, but found no polymerization activity under our standard polymerization conditions for all five amine/LA systems. To gain further insight into this observation, we carried out NMR scale reactions for the amine/LA pairs with equimolar MMA (Figure 3). Accordingly, upon mixing stoichiometric amounts of Et_3_N/B(C_6_F_5_)_3_/MMA, only the signals of MMA and the products of the disproportionation reaction of Et_3_N/B(C_6_F_5_)_3_ were observed, regardless of the mixing sequence. Interestingly, addition of more B(C_6_F_5_)_3_ into the above mixture resulted in the consumption of MMA and formation of a new species, which was identified as methyl isobutyrate, Me_2_CHC(=O)OMe, a product of C=C double bond hydrogenation of MMA (Scheme 3). This transfer hydrogenation was fast and clean, and all of the MMA was consumed in the presence of an additional equivalent of B(C_6_F_5_)_3_. Noteworthy is the inability to generate the ammonium enolborate initiation species, which was further supported by the presence of the geminal protons (=CH_2_) NMR signals derived from a 1:1:1 mixture of TMP/B(C_6_F_5_)_3_/MMA (see Supporting Information). Similarly, mixing of a preformed Et_3_N·Al(C_6_F_5_)_3_ adduct with MMA only resulted in the replacement of Et_3_N with MMA, as indicated by the generation of free Et_3_N and the remaining geminal protons (=CH_2_) signals. Overall, these NMR experiments showed that the combination of Et_3_N or TMP with E(C_6_F_5_)_3_ provides insufficient activation for the polymerization of MMA, either due to the reduction of MMA into the inactive methyl isobutyrate, or the steric bulkiness around the amine, which impairs the effective formation of the active species for polymerization.

### 2.3. Characteristic of _γ_MMBL Polymerization by Amine/E(C_6_F_5_)_3_

Next, we set out to investigate activity of the amine/E(C_6_F_5_)_3_ systems toward cyclic _γ_MMBL, the results of which were summarized in Table 1. The intramolecular FLP system **5** yielded no polymer formation up to 24 h, under the current standard conditions and with or without another equivalent of B(C_6_F_5_)_3_ (entries 17 and 18). Mixing of equimolar _γ_MMBL and **5** in CD_2_Cl_2_ led to formation of adduct _γ_MMBL·**5** via the Lewis acidic borane site in **5**, but no active species or polymer formation. This result implies that the inability of **5** to polymerize _γ_MMBL is attributed to the lack of initiation by the bulky amine site in **5**. On the other hand, the intermolecular Lewis pair systems **1** to **4** initiated rapid and quantitative polymerization of *_γ_*MMBL into P_γ_MMBL. Under our current standard polymerization conditions ([_γ_MMBL]:[LA]:[LB] = 200:2:1, 0.5 mL _γ_MMBL, 2.0 mL CH_2_Cl_2_, RT), Et_3_N/LA system **1** (B) or **3** (Al) polymerized _γ_MMBL with full conversion in 1 or 0.5 min, giving P_γ_MMBL with *M*_n_ = 3.12 or 1.92 × 10^4^ g∙mol^−1^ (*Đ* = 2.31 or 2.02), thus achieving an averaged turn-over frequency (TOF) of 12,000 or 24,000 h^−1^ for the borane or alane, respectively (entries 1 and 3). Increasing the ratio to 800:2:1 resulted in an increase of *M*_n_ to 5.13 or 3.30 × 10^4^ g∙mol^−1^ for the B and Al LA, respectively, although a significantly prolonged polymerization period of 60 or 30 min was needed to achieve full monomer conversion (entries 2 and 4). Overall, the alane showed two-fold higher activity over the borane when coupled with Et_3_N. Moreover, the reverse addition sequence, in which equimolar Et_3_N and LA were premixed, followed by addition to a CH_2_Cl_2_ solution of _γ_MMBL, was also effective, albeit a slightly slower rate compared to the standard procedure, with or without an additional equivalent of LA. A stoichiometric reaction of Et_3_N/B(C_6_F_5_)_3_/_γ_MMBL revealed that all of the monomer was rapidly consumed, with generation of P_γ_MMBL (see Supporting Information). We reasoned that ammonium hydridoborate, formed by premixing of Et_3_N and B(C_6_F_5_)_3_, can initiate the polymerization via the nucleophilic hydride, whereas the Et_3_N·Al(C_6_F_5_)_3_ adduct formed via premixing can dissociate in the presence of _γ_MMBL and thus promote the subsequent initiation and bimolecular, activated monomer propagation.

As mentioned above, the combination of the alane or the borane with TMP offered more well-defined coordination chemistry (*i.e*., either CLA or FLP). Hence, the TMP/LA pairs were investigated for LPP with 6 different [monomer]:[LA]:[TMP] ratios, ranging from 50:2:1 to 1600:2:1, as well as different addition sequences. Impressively, the polymerizations were finished within 1 min for a ratio up to of 800:2:1 (entries 5–9 and 11–15), and even under lower catalyst loading (0.0625 mol % relative to TMP) conditions (1600:2:1), the polymerizations were completed in 3 min (entries 10 and 16). Thus, the polymerization by the B/TMP and Al/TMP Lewis pairs was very rapid with TOF up to 96,000 h^−1^ (for Al), providing polymers with *M*_n_ ranging from medium 6.75 × 10^4^ to high 2.55 × 10^5^ g∙mol^−1^ (*Đ* = 1.54 to 1.92) and medium 6.96 × 10^4^ to high 1.38 × 10^5^ g∙mol^−1^ (*Đ* = 2.16 to 2.42) for the borane and alane, respectively. Interestingly, under the current standard conditions, if the LAs were premixed with TMP and added to a CH_2_Cl_2_ solution of the monomer, the polymerization proceeded rapidly as well. This result confirmed that the TMP·Al(C_6_F_5_)_3_ adduct formed via premixing can rapidly dissociate in the presence of _γ_MMBL monomer and promote the subsequent initiation and bimolecular, activated monomer propagation [58,59], while the B/TMP FLP should be active regardless of the addition sequence.

**Table 1:** Selected results of polymerization of _γ_MMBL by LA/amine LPs ^a^. 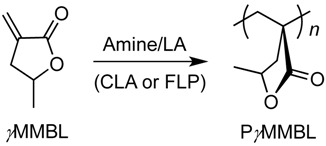

Entry	LP	[M]/[Base]	Time (min)	Conv. ^b^ (%)	TOF (h^-1^)	*M*_n_^c^ (kg∙mol^−1^)	*Đ* ^d^ (*M*_w_/*M*_n_)
1	B/Et_3_N (**1**)	200	1	100	12,000	31.2	2.31
2		800	60	100	800	53.1	2.47
3	Al/Et_3_N (**3**)	200	0.5	100	24,000	19.2	2.02
4		800	30	100	1600	33.0	2.26
5	B/TMP (**2**)	50	0.5	100	6000	67.5 ^d^	1.54
6		100	0.5	100	12,000	100 ^d^	1.64
7		200	0.5	100	24,000	145 ^d^	1.78
8		400	0.5	100	48,000	156 ^d^	1.92
9		800	1	100	48,000	253 ^d^	1.64
10		1600	3	100	32,000	255 ^d^	1.66
11	Al/TMP (**4**)	50	0.5	100	6000	69.6	2.41
12		100	0.5	100	12,000	77.2	2.29
13		200	0.5	100	24,000	113	2.16
14		400	0.5	100	48,000	119	2.38
15		800	0.5	100	96,000	129	2.21
16		1600	3	100	32,000	138	2.16
17	B-TMP (**5**)	200	1440	0	-	-	-
18 ^e^	**5** + B	200	1440	0	-	-	-

^a^ Conditions: carried out at RT (*ca*. 25 °C) in CH_2_Cl_2_ with 2.5 mL of the total solution; ^b^ Monomer conversion measured by ^1^H-NMR spectroscopy; ^c^ Number-average molecular weight (*M*_n_) and molecular weight distribution (*Đ*) determined by GPC in DMF relative to PMMA standards; ^d^ Bimodal distribution, with an additional small peak (5.0% to 18.3%) on the higher molecular weight region; ^e^ Carried out with an additional equivalent of B(C_6_F_5_)_3_.

## 3. Experimental Section

### 3.1. Materials, Reagents, and Methods

All syntheses and manipulations of air- and moisture-sensitive materials were carried out in flamed Schlenk-type glassware on a dual-manifold Schlenk line, on a high-vacuum line, or in an inert gas-filled glovebox. NMR-scale reactions were conducted in Teflon-valve-sealed J. Young-type NMR tubes. HPLC-grade organic solvents were first sparged extensively with nitrogen during filling 20 L solvent reservoirs and then dried by passage through activated alumina (for Et_2_O, THF, and CH_2_Cl_2_) followed by passage through Q-5 supported copper catalyst (for toluene and hexanes) stainless steel columns. Benzene-*d*_6_ and toluene-*d*_8_ were dried over sodium/potassium alloy and vacuum-distilled or filtered, whereas CD_2_Cl_2_ and CDCl_3_ were dried over activated Davison 4 Å molecular sieves. HPLC-grade dimethyl formamide (DMF) was degassed and dried over CaH_2_ overnight, followed by vacuum distillation (CaH_2_ was removed before distillation). NMR spectra were recorded on Varian Inova 300 (300 MHz, ^1^H; 75 MHz, ^13^C; 282 MHz, ^19^F) or a Varion 400 MHz spectrometer. Chemical shifts for ^1^H-, ^19^F- and ^13^C- spectra were referenced to internal solvent resonances and are reported as parts per million relative to SiMe_4_.

Methyl methacrylate (MMA) was purchased from Sigma-Aldrich Co. (St. Louis, MO, USA), while γ-methyl-α-methylene-γ-butyrolactone (*_γ_*MMBL) was purchased from TCI America (Portland, OR, USA). These monomers were first degassed and dried over CaH_2_ overnight, followed by vacuum distillation. Further purification of MMA involved titration with neat tri(*n*-octyl)aluminum to a yellow end point [76], followed by distillation under reduced pressure. All purified monomers were stored in brown bottles and stored inside a glovebox freezer at −30 °C. Amines including triethylamine and 2,2,6,6-tetramethylpiperidine (TMP) were purchased from Sigma-Aldrich Co (St. Louis, MO, USA), distilled over CaH_2_, and store brown bottles in a glovebox prior to use. BHT-H was recrystallized from hexanes prior to use. Tris(pentafluorophenyl)borane, obtained as a research gifts from Boulder Scientific Company (Mead, CO, USA), was further purified by sublimation under vacuum. Tris(pentafluorophenyl)alane·(toluene)_0.5_ [77,78,79] and 1-(2-[bis(pentafluorophenyl)boryl]benzyl)-2,2,6,6-tetramethylpiperidine [67] were synthesized according to literature procedures. 

### 3.2. Isolation of Adduct Et_3_N·Al(C_6_F_5_)_3_

Al(C_6_F_5_)_3_·(toluene)_0.5_ (135 mg, 0.235 mmol) and Et_3_N (32.6 μL, 0.235 mmol, 1.0 equiv) were dissolved in toluene (1 mL) in a glovebox. The mixture was allowed to react for 10 min, then layered with hexanes (4 mL), and placed in a freezer and recrystallized at −35 °C. The titled product was isolated as colorless crystals. Yield: 120 mg (81%). ^1^H-NMR (C_6_D_6_, 23 °C): δ 2.60 (q, *J* = 7.2 Hz, 6H, C*H_2_*CH_3_), 0.55 (t, 9H, CH_2_C*H*_3_). ^13^C-NMR (C_6_D_6_, 23 °C): δ 150.1, 141.9, 137.4 (C_6_F_5_, *ipso*-C_6_F_5_-Al not observed), 47.4 (*C*H_2_), 8.8 (*C*H_3_). ^19^F-NMR (C_6_D_6_, 23 °C): δ −119.0 (d, ^3^*J* = 19.4 Hz, 6F, *o*-F), −152.0 (t, ^3^*J* = 19.9 Hz, 3F, *p*-F), −160.9 (m, 6F, *m*-F) ppm.

### 3.3. Isolation of Adduct TMP·Al(C_6_F_5_)_3_

Al(C_6_F_5_)_3_·(toluene)_0.5_ (141 mg, 0.246 mmol) and TMP (42 μL, 0.246 mmol, 1.0 equiv) were dissolved in toluene (1 mL) in a glovebox. The mixture was allowed to react for 10 min, then layered with hexanes (3 mL), and placed in a freezer and recrystallized at −35 °C. The titled product was isolated as colorless crystals and kept in the freezer to avoid decomposition. Yield: 140 mg (85%). ^1^H-NMR (C_6_D_6_, 23 °C): δ 3.72 (br, 1H, N*H*), 1.09 (br, 6H, C*H*_3_), 0.93 (br, 6H, C*H_3_*), 0.90 (br, 4H, C*H_2_*), 0.75 (m, 2H, C*H_2_*). ^13^C-NMR (C_6_D_6_, 23 °C): δ 150.2, 142.1, 137.5 (C_6_F_5_, ipso-C_6_F_5_-Al not observed), 60.4, 40.6, 32.7, 24.7, 16.2 (TMP). ^19^F-NMR (C_6_D_6_, 23 °C): δ −119.1 (d, ^3^*J* = 16.1 Hz, 6F, *o*-F), −151.7 (t, ^3^*J* = 20.3 Hz, 3F, *p*-F), −161.2 (pst, ^3^*J* = 18.3 Hz, 6F, *m*-F) ppm.

### 3.4. Stoichiometric Hydrogenation of MMA by Et_3_N/2B(C_6_F_5_)_3_ to Methyl Isobutyrate

A CD_2_Cl_2_ (0.7 mL) solution of MMA (5.0 L, 0.0469 mmol) in a J. Young NMR tube was added B(C_6_F_5_)_3_ (24 mg, 0.0469 mmol), followed by addition of Et_3_N (6.54 L, 0.0469 mmol). The mixture was allowed to react for 10 min before ^1^H-NMR measurement, which indicated formation of ammonium hydridoborate (C_6_F_5_)_3_B-H···H-N(C_2_H_5_)_3_ and iminium zwitterion (C_6_F_5_)_3_B-CH_2_-CH=N(C_2_H_5_)_2_ but without consuming any MMA (Scheme 3, Figure 3). In the next step, a second equivalent of B(C_6_F_5_)_3_ was transferred to the same NMR tube and the reaction progress was monitored by ^1^H-NMR. The transfer hydrogenation went to completion yielding methyl isobutyrate.

### 3.5. Typical Procedure for the NMR Scale Reactions (TMP/B(C_6_F_5_)_3_/MMA as an Example)

A CD_2_Cl_2_ (0.7 mL) solution of MMA (5.0 L, 0.0469 mmol) in a J. Young NMR tube was added B(C_6_F_5_)_3_ (24 mg, 0.0469 mmol), followed by addition of TMP (7.98 L, 0.0469 mmol). The mixture was allowed to react for 10 min before ^1^H-NMR measurement, which indicated the retention of the geminal protons of MMA.

In the case of the alane, a different addition sequence was adopted, in which the preformed amine/Al adduct was transferred to the corresponding monomer in specific deuterated solvents as the solvent will decompose the alane upon mixing directly.

### 3.6. General Polymerization Procedures

Polymerizations were performed either in 25 mL flame-dried Schlenk flasks interfaced to the dual-manifold Schlenk line for runs using external temperature bath, or in 20 mL glass reactors inside the glovebox for ambient temperature (*ca.* 25 °C) runs. In a typical polymerization procedure, a predetermined amount of B(C_6_F_5_)_3_ or Al(C_6_F_5_)_3_ was first dissolved in a monomer (0.5 mL of MMA or _γ_MMBL, 200 equiv relative to the LB) and 2.0 mL of solvent (CH_2_Cl_2_) inside a glovebox. The polymerization was started by rapid addition of a solution of a LB (1 equiv of an amine) in 0.5 mL of CH_2_Cl_2_ via a gastight syringe to the above mixture containing the LA and monomer under vigorous stirring. The amount of the monomer was fixed for the varied [M]/[LB] ratio runs. In a second procedure of polymerization, the premixed LA/LB system (or the intramolecular Lewis pair) was dissolved in 0.5 mL of toluene (for the alane system since the alane is known to decompose in CH_2_Cl_2_ in the absence of a monomer) or CH_2_Cl_2_ (for the borane system), followed by addition to a CH_2_Cl_2_ (2.0 mL) solution of monomer (0.5 mL) to initiate the polymerization. After the measured time interval, a 0.2 mL aliquot was taken from the reaction mixture via syringe and quickly quenched into a 4-mL vial containing 0.6 mL of undried “wet” CDCl_3_ stabilized by 250 ppm of BHT-H; the quenched aliquots were later analyzed by ^1^H-NMR to obtain the percent monomer conversion data. After the polymerization was stirred for the stated reaction time and then the polymer was immediately precipitated into 200 mL of methanol, stirred for 1 h, filtered, washed with methanol, and dried in a vacuum oven at 50 °C overnight to a constant weight.

### 3.7. Polymer Characterizations

Polymer number-average molecular weights (*M*_n_) and molecular weight distributions (*Đ* = *M*_w_/*M*_n_) were measured by gel permeation chromatography (GPC) analyses carried out at 40 °C and a flow rate of 1.0 mL∙min^−1^, with DMF as the eluent, on a Waters University 1500 GPC instrument equipped with one PLgel 5 μm guard and three PLgel 5 μm mixed-C columns (Polymer Laboratories; linear range of molecular weight = 200–2,000,000). The instrument was calibrated with 10 PMMA standards, and chromatograms were processed with Waters Empower software (version 2002).

## 4. Conclusions

In summary, Lewis pairs consisting of bulky amine Et_3_N and TMP as LBs and bulky E(C_6_F_5_)_3_ as strong organo-LAs were employed for investigation into their reactivity toward two acrylic monomers, including linear MMA and cyclic *_γ_*MMBL. While TMP and B(C_6_F_5_)_3_ form an FLP, Et_3_N reacts with B(C_6_F_5_)_3_ to form disproportionation products ammonium hydridoborate ionic pair and iminium zwitterion. On the other hand, the stoichiometric reaction of Et_3_N and TMP with Al(C_6_F_5_)_3_ leads to clean formation CLAs.

In the case of linear MMA, the Et_3_N/2B(C_6_F_5_)_3_ pair promotes transfer hydrogenation of MMA to form methyl isobutyrate. The TMP/B(C_6_F_5_)_3_ FLP is also incapable of polymerizing MMA, attributable to the steric hindrance and low nucleophilicity at N in TMP, which resulted in no formation of the active propagating species. In contrast, the amine/LA pairs promote rapid polymerization of _γ_MMBL carrying the more reactive exocyclic methylene moiety, achieving full conversion in less than 3 min even at a low catalyst loading of 0.0625 mol %. The Al/Et_3_N pair displays higher activity when compared with the B/Et_3_N pair. Meanwhile, TMP is more efficient than Et_3_N when paired with either the borane or the alane. Of the four LA/LB pairs investigated in this study, the TMP/Al(C_6_F_5_)_3_ pair exhibits the highest activity, achieving a maximum TOF of 96,000 h^−1^ at 0.125 mol % of catalyst loading or 32,000 h^−1^ at 0.0625 mol %, producing high molecular weight P_γ_MMBL with *M*_n_ = 1.29 × 10^5^ or 1.38 × 10^5^ g∙mol^−1^, respectively. As compared with the previous LBs employed for LPP, such as phosphines and NHCs, the current LPP system utilizing the inexpensive, readily available and more stable and environmentally friendly amine LBs will provide a greener alternative approach toward bio-renewable polymers based on _γ_MMBL and other monomers.

## Supplementary Materials

Supplementary materials can be accessed at: http://www.mdpi.com/1420-3049/20/06/9575/s1.

## References

[1] Stephan D.W. (2015). Frustrated Lewis pairs: From concept to catalysis. Acc. Chem. Res..

[2] Stephan D.W., Erker G. (2014). Frustrated Lewis pair chemistry of carbon, nitrogen and sulfur oxides. Chem. Sci..

[3] Erker G., Stephan D.W. (2013). Frustrated Lewis Pairs I & II.

[4] Stephan D.W. (2012). “Frustrated Lewis pair” hydrogenations. Org. Biomol. Chem..

[5] Erker G. (2012). Frustrated Lewis pairs: Some recent developments. Pure Appl. Chem..

[6] Erker G. (2011). Organometallic frustrated Lewis pair chemistry. Dalton Trans..

[7] Stephan D.W., Erker G. (2010). Frustrated Lewis pairs: Metal-free hydrogen activation and more. Angew. Chem. Int. Ed..

[8] Stephan D.W. (2009). Frustrated Lewis pairs: A new strategy to small molecule activation and hydro-genation catalysis. Dalton Trans..

[9] Stephan D.W. (2008). “Frustrated Lewis pairs”: A concept for new reactivity and catalysis. Org. Biomol. Chem..

[10] Lawrence E.J., Oganesyan V.S., Hughes D.L., Ashley A.E., Wildgoose G.G. (2014). An electrochemical study of frustrated Lewis pairs: A metal-free route to hydrogen oxidation. J. Am. Chem. Soc..

[11] Sajid M., Elmer L.M., Rosorius C., Daniliuc C.G., Grimme S., Kehr G., Erker G. (2013). Facile carbon monoxide reduction at intramolecular frustrated phosphane/borane Lewis pair templates. Angew. Chem. Int. Ed..

[12] Dobrovetsky R., Stephan D.W. (2013). Stoichiometric metal-free reduction of CO in syn-gas. J. Am. Chem. Soc..

[13] Appelt C., Slootweg J.C., Lammertsma K., Uhl W. (2013). Reaction of a P/Al-based frustrated Lewis pair with ammonia, borane, and amine-boranes: Adduct formation and catalytic dehydrogenation. Angew. Chem. Int. Ed..

[14] Bertini F., Lyaskoyskyy V., Timmer B.J.J., de Kanter F.J.J., Lutz M., Ehlers A.W., Slootweg J.C., Lammertsma K. (2012). Preorganized frustrated Lewis pairs. J. Am. Chem. Soc..

[15] Schafer A., Reissmann M., Schafer A., Saak W., Haase D., Muller T. (2011). A new synthesis of triarylsilylium ions and their application in dihydrogen activation. Angew. Chem. Int. Ed..

[16] Marwitz A.J.V., Dutton J.L., Mercier L.G., Piers W.E. (2011). Dihydrogen activation with ^*t*^Bu_3_P/B(C_6_F_5_)_3_: A chemically competent indirect mechanism via in situ-generated *p*-^*t*^Bu_2_P-C_6_F_4_-B(C_6_F_5_)_2_. J. Am. Chem. Soc..

[17] Lu Z.P., Cheng Z.H., Chen Z.X., Weng L.H., Li Z.H., Wang H.D. (2011). Heterolytic cleavage of dihydrogen by “frustrated Lewis pairs” comprising bis(2,4,6-tris(trifluoromethyl)phenyl)borane and amines: Stepwise *versus* concerted mechanism. Angew. Chem. Int. Ed..

[18] Ekkert O., Kehr G., Frohlich R., Erker G. (2011). P-C bond activation chemistry: Evidence for 1,1-carboboration reactions proceeding with phosphorus-carbon bond cleavage. J. Am. Chem. Soc..

[19] Appelt C., Westenberg H., Bertini F., Ehlers A.W., Slootweg J.C., Lammertsma K., Uhl W. (2011). Geminal phosphorus/aluminum-based frustrated Lewis pairs: C-H *versus* C equivalent to C activation and CO_2_ fixation. Angew. Chem. Int. Ed..

[20] Ines B., Holle S., Goddard R., Alcarazo M. (2010). Heterolytic S-S bond cleavage by a purely carbogenic frustrated Lewis pair. Angew. Chem. Int. Ed..

[21] Grimme S., Kruse H., Goerigk L., Erker G. (2010). The mechanism of dihydrogen activation by frustrated Lewis pairs revisited. Angew. Chem. Int. Ed..

[22] Momming C.M., Otten E., Kehr G., Frohlich R., Grimme S., Stephan D.W., Erker G. (2009). Reversible metal-free carbon dioxide binding by frustrated Lewis pairs. Angew. Chem. Int. Ed..

[23] Holschumacher D., Bannenberg T., Hrib C.G., Jones P.G., Tamm M. (2008). Heterolytic dihydrogen activation by a frustrated carbene-borane Lewis pair. Angew. Chem. Int. Ed..

[24] Chase P.A., Stephan D.W. (2008). Hydrogen and amine activation by a frustrated Lewis pair of a bulky *N*-heterocyclic carbene and B(C_6_F_5_)_3_. Angew. Chem. Int. Ed..

[25] Sumerin V., Schulz F., Nieger M., Leskelä M., Repo T., Rieger B. (2008). Facile heterolytic H_2_ activation by amines and B(C_6_F_5_)_3_. Angew. Chem. Int. Ed..

[26] Hounjet L.J., Bannwarth C., Garon C.N., Caputo C.B., Grimme S., Stephan D.W. (2013). Combinations of ethers and B(C_6_F_5_)_3_ function as hydrogenation catalysts. Angew. Chem. Int. Ed..

[27] Greb L., Daniliuc C.G., Bergander K., Paradies J. (2013). Functional-group tolerance in frustrated Lewis pairs: Hydrogenation of nitroolefins and acrylates. Angew. Chem. Int. Ed..

[28] Chernichenko K., Madarasz A., Papai I., Nieger M., Leskela M., Repo T. (2013). A frustrated-Lewis-pair approach to catalytic reduction of alkynes to cis-alkenes. Nat. Chem..

[29] Miller A.J.M., Labinger J.A., Bercaw J.E. (2010). Homogeneous CO hydrogenation: Dihydrogen activation involves a frustrated Lewis pair instead of a platinum complex. J. Am. Chem. Soc..

[30] Eros G., Mehdi H., Papai I., Rokob T.A., Kiraly P., Tarkanyi G., Soos T. (2010). Expanding the scope of metal-free catalytic hydrogenation through frustrated Lewis pair design. Angew. Chem. Int. Ed..

[31] Axenov K.V., Kehr G., Frohlich R., Erker G. (2009). Catalytic hydrogenation of sensitive organometallic compounds by antagonistic N/B Lewis pair catalyst systems. J. Am. Chem. Soc..

[32] Ashley A.E., Thompson A.L., O’Hare D. (2009). Non-metal-mediated homogeneous hydrogenation of CO_2_ to CH_3_OH. Angew. Chem. Int. Ed..

[33] Wang X.W., Kehr G., Daniliuc C.G., Erker G. (2014). Internal adduct formation of active intramolecular C-4-bridged frustrated phosphane/borane Lewis pairs. J. Am. Chem. Soc..

[34] Rocchigiani L., Ciancaleoni G., Zuccaccia C., Macchioni A. (2014). Probing the association of frustrated phosphine-borane Lewis pairs in solution by NMR spectroscopy. J. Am. Chem. Soc..

[35] Henthorn J.T., Agapie T. (2014). Dioxygen reactivity with a ferrocene-Lewis acid pairing: Reduction to a boron peroxide in the presence of tris(pentafluorophenyl)borane. Angew. Chem. Int. Ed..

[36] Sajid M., Kehr G., Wiegand T., Eckert H., Schwickert C., Pottgen R., Cardenas A.J.P., Warren T.H., Frohlich R., Daniliuc C.G. (2013). Noninteracting, vicinal frustrated P/B-Lewis pair at the norbornane framework: Synthesis, characterization, and reactions. J. Am. Chem. Soc..

[37] Menard G., Hatnean J.A., Cowley H.J., Lough A.J., Rawson J.M., Stephan D.W. (2013). C-H bond activation by radical ion pairs derived from R_3_P/Al(C_6_F_5_)_3_ frustrated Lewis pairs and N_2_O. J. Am. Chem. Soc..

[38] Zhao X.X., Stephan D.W. (2011). Olefin-borane “van der Waals complexes”: Intermediates in frustrated Lewis pair addition reactions. J. Am. Chem. Soc..

[39] Menard G., Stephan D.W. (2011). Stoichiometric reduction of CO_2_ to CO by aluminum-based frustrated Lewis pairs. Angew. Chem. Int. Ed..

[40] Chapman A.M., Haddow M.F., Wass D.F. (2011). Frustrated Lewis pairs beyond the main group: Cationic zirconocene-phosphinoaryloxide complexes and their application in catalytic dehydrogenation of amine boranes. J. Am. Chem. Soc..

[41] Cardenas A.J.P., Culotta B.J., Warren T.H., Grimme S., Stute A., Frohlich R., Kehr G., Erker G. (2011). Capture of NO by a frustrated Lewis pair: A new type of persistent N-oxyl radical. Angew. Chem. Int. Ed..

[42] Momming C.M., Kehr G., Wibbeling B., Frohlich R., Schirmer B., Grimme S., Erker G. (2010). Formation of cyclic allenes and cumulenes by cooperative addition of frustrated Lewis pairs to conjugated enynes and diynes. Angew. Chem. Int. Ed..

[43] Menard G., Stephan D.W. (2010). Room temperature reduction of CO_2_ to methanol by Al-based frustrated Lewis pairs and ammonia borane. J. Am. Chem. Soc..

[44] Berkefeld A., Piers W.E., Parvez M. (2010). Tandem frustrated Lewis pair/tris(pentafluorophenyl) borane-catalyzed deoxygenative hydrosilylation of carbon dioxide. J. Am. Chem. Soc..

[45] Alcarazo M., Gomez C., Holle S., Goddard R. (2010). Exploring the reactivity of carbon(0)/borane-based frustrated Lewis pairs. Angew. Chem. Int. Ed..

[46] Lindqvist M., Borre K., Axenov K., Kótai B., Nieger M., Leskelä M., Pápai I., Repo T. (2015). Chiral molecular tweezers: Synthesis and reactivity in asymmetric hydrogenation. J. Am. Chem. Soc..

[47] Wei S.M., Du H.F. (2014). A highly enantioselective hydrogenation of silyl enol ethers catalyzed by chiral frustrated Lewis pairs. J. Am. Chem. Soc..

[48] Chen J., Lalancette R.A., Jäkle F. (2014). Chiral organoborane Lewis pairs derived from pyridylferrocene. Chem. Eur. J..

[49] Liu Y.B., Du H.F. (2013). Chiral dienes as “ligands” for borane-catalyzed metal-free asymmetric hydrogenation of imines. J. Am. Chem. Soc..

[50] Chen J., Lalancette R.A., Jäkle F. (2013). Synthesis and Lewis acid properties of a ferrocene-based planar-chiral borenium cation. Chem. Commun..

[51] Ghattas G., Chen D.J., Pan F.F., Klankermayer J. (2012). Asymmetric hydrogenation of imines with a recyclable chiral frustrated Lewis pair catalyst. Dalton Trans..

[52] Sumerin V., Chernichenko K., Nieger M., Leskela M., Rieger B., Repo T. (2011). Highly active metal-free catalysts for hydrogenation of unsaturated nitrogen-containing compounds. Adv. Synth. Catal..

[53] Chen J., Venkatasubbaiah K., Pakkirisamy T., Doshi A., Yusupov A., Patel Y., Lalancette R.A., Jäkle F. (2010). Planar chiral organoborane Lewis acids derived from naphthylferrocene. Chem. Eur. J..

[54] Chen D.J., Wang Y.T., Klankermayer J. (2010). Enantioselective hydrogenation with chiral frustrated Lewis pairs. Angew. Chem. Int. Ed..

[55] Chen D.J., Klankermayer J. (2008). Metal-free catalytic hydrogenation of imines with tris(perfluorophenyl)borane. Chem. Commun..

[56] Chen E.Y.X. (2013). Polymerization by classical and frustrated Lewis pairs. Top. Curr. Chem..

[57] Chen J., Chen E.Y.X. (2015). Lewis pair polymerization of acrylic monomers by *N*-heterocyclic carbenes and B(C_6_F_5_)_3_. Isr. J. Chem..

[58] Zhang Y.T., Miyake G.M., John M.G., Falivene L., Caporaso L., Cavallo L., Chen E.Y.X. (2012). Lewis pair polymerization by classical and frustrated Lewis pairs: Acid, base and monomer scope and polymerization mechanism. Dalton Trans..

[59] Zhang Y.T., Miyake G.M., Chen E.Y.X. (2010). Alane-based classical and frustrated Lewis pairs in polymer synthesis: Rapid polymerization of MMA and naturally renewable methylene butyrolactones into high-molecular-weight polymers. Angew. Chem. Int. Ed..

[60] He J.H., Zhang Y.T., Falivene L., Caporaso L., Cavallo L., Chen E.Y.X. (2014). Chain propagation and termination mechanisms for polymerization of conjugated polar alkenes by [Al]-Based frustrated Lewis pairs. Macromolecules.

[61] He J.H., Zhang Y.T., Chen E.Y.X. (2014). Synthesis of pyridine- and 2-oxazoline-functionalized vinyl polymers by alane-based frustrated Lewis pairs. Synlett.

[62] Piedra-Arroni E., Ladaviere C., Amgoune A., Bourissou D. (2013). Ring-opening polymerization with Zn(C_6_F_5_)_2_-based Lewis pairs: Original and efficient approach to cyclic polyesters. J. Am. Chem. Soc..

[63] Sajid M., Stute A., Cardenas A.J.P., Culotta B.J., Hepperle J.A.M., Warren T.H., Schirmer B., Grimme S., Studer A., Daniliuc C.G. (2012). *N*,*N*-Addition of frustrated Lewis pairs to nitric oxide: An easy entry to a unique family of aminoxyl radicals. J. Am. Chem. Soc..

[64] Xu T.Q., Chen E.Y.X. (2014). Probing site cooperativity of frustrated phosphine/borane Lewis pairs by a polymerization study. J. Am. Chem. Soc..

[65] Jia Y.B., Ren W.M., Liu S.J., Xu T.Q., Wang Y.B., Lu X.B. (2014). Controlled divinyl monomer polymerization mediated by Lewis pairs: A powerful synthetic strategy for functional polymers. ACS Macro Lett..

[66] Eisenberger P., Bailey A.M., Crudden C.M. (2012). Taking the F out of FLP: Simple Lewis acid-base pairs for mild reductions with neutral boranes via borenium ion catalysis. J. Am. Chem. Soc..

[67] Sumerin V., Schulz F., Atsumi M., Wang C., Nieger M., Leskela M., Repo T., Pyykko P., Rieger B. (2008). Molecular tweezers for hydrogen: Synthesis, characterization, and reactivity. J. Am. Chem. Soc..

[68] Schulz F., Sumerin V., Heikkinen S., Pedersen B., Wang C., Atsumi M., Leskela M., Repo T., Pyykko P., Petry W. (2011). Molecular hydrogen tweezers: Structure and mechanisms by neutron diffraction, NMR, and deuterium labeling studies in solid and solution. J. Am. Chem. Soc..

[69] Mountford A.J., Lancaster S.J., Coles S.J., Horton P.N., Hughes D.L., Hursthouse M.B., Light M.E. (2005). Intra- and intermolecular N-H···F-C hydrogen-bonding interactions in amine adducts of tris(pentafluorophenyl)borane and -alane. Inorg. Chem..

[70] Ning Y., Zhu H.P., Chen E.Y.X. (2007). Remarkable Lewis acid effects on polymerization of functionalized alkenes by metallocene and lithium ester enolates. J. Organomet. Chem..

[71] Rodriguez-Delgado A., Chen E.Y.X. (2005). Single-site anionic polymerization. Monomeric ester enolaluminate propagator synthesis, molecular structure, and polymerization mechanism. J. Am. Chem. Soc..

[72] Chen E.Y.X., Kruper W.J., Roof G., Wilson D.R. (2001). “Double activation” of constrained geometry and ansa-metallocene group 4 metal dialkyls: Synthesis, structure, and olefin polymerization study of mono-and dicationic aluminate complexes. J. Am. Chem. Soc..

[73] Bolig A.D., Chen E.Y.X. (2001). Reversal of polymerization stereoregulation in anionic polymerization of MMA by chiral metallocene and non-metallocene initiators: A new reaction pathway for metallocene-initiated MMA polymerization. J. Am. Chem. Soc..

[74] Chen J., Chen E.Y.X. (2015). Elusive silane alane complex [Si-H···Al]: Isolation, characterization, and multifaceted frustrated-Lewis-pair-type catalysis. Angew. Chem. Int. Ed..

[75] Di Saverio A., Focante F., Camurati I., Resconi L., Beringhelli T., D’Alfonso G., Donghi D., Maggioni D., Mercandelli P., Sironi A. (2005). Oxygen-bridged borate anions from tris(pentafluorophenyl)borane: Synthesis, NMR characterization, and reactivity. Inorg. Chem..

[76] Allen R.D., Long T.E., Mcgrath J.E. (1986). Preparation of high-purity, anionic-polymerization grade alkyl methacrylate monomers. Polym. Bull..

[77] Feng S.G., Roof G.R., Chen E.Y.X. (2002). Tantalum (V)-based metallocene, half-metallocene, and non-metallocene complexes as ethylene-1-octene copolymerization and methyl methacrylate polymerization catalysts. Organometallics.

[78] Lee C.H., Lee S.J., Park J.W., Kim K.H., Lee B.Y., Oh J.S. (1998). Preparation of Al(C_6_F_5_)_3_ and its use for the modification of methylalumoxane. J. Mol. Catal. A Chem..

[79] Biagini P., Lugli G., Abis L., Andreussi P. (1997). Organometallic derivatives of group IIIA and process for their preparation. U.S. Patent.

