# Supplementary material for: Reactivity of Amine/E(C6F5)3 (E = B, Al) Lewis Pairs toward Linear and Cyclic Acrylic Monomers: Hydrogenation vs. Polymerization
Source: Molecules. 2015 May 26;20(6):9575–90. doi: 10.3390/molecules20069575 (PMC6272166; doi:10.3390/molecules20069575)
Supplement: Supplementary file 1 [file molecules-20-09575-s001.pdf]

Supplementary Information

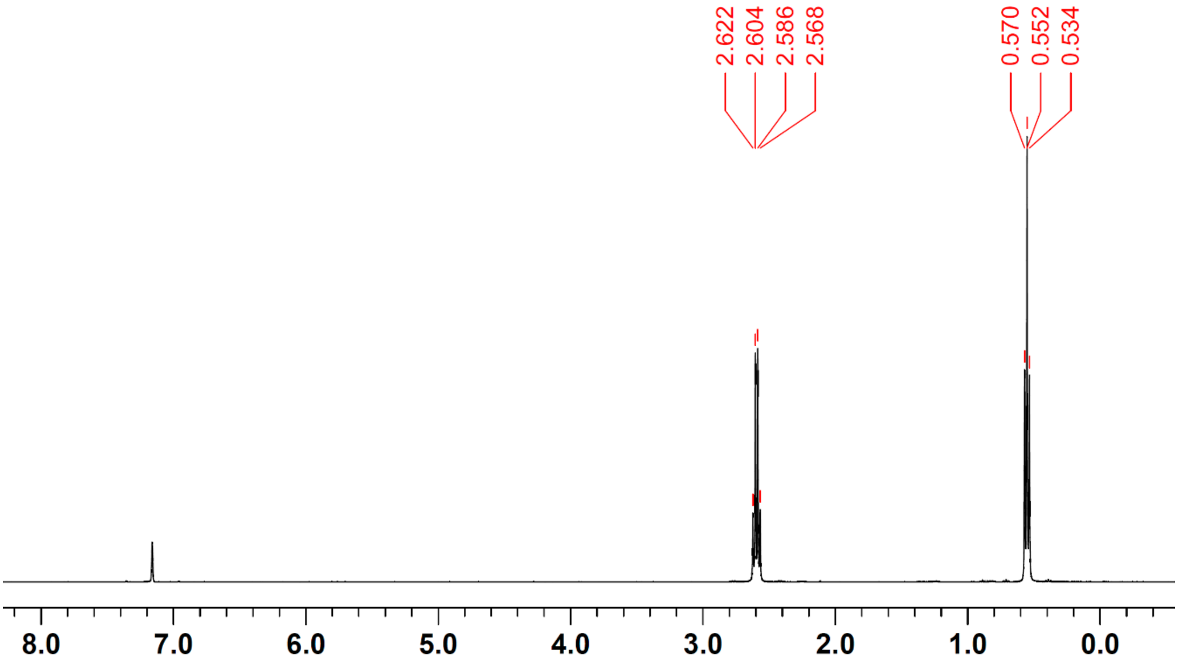

Figure S1. ^{1}H-NMR spectrum of adduct $Et_3N{\cdot}Al(C_6F_5)_3$.

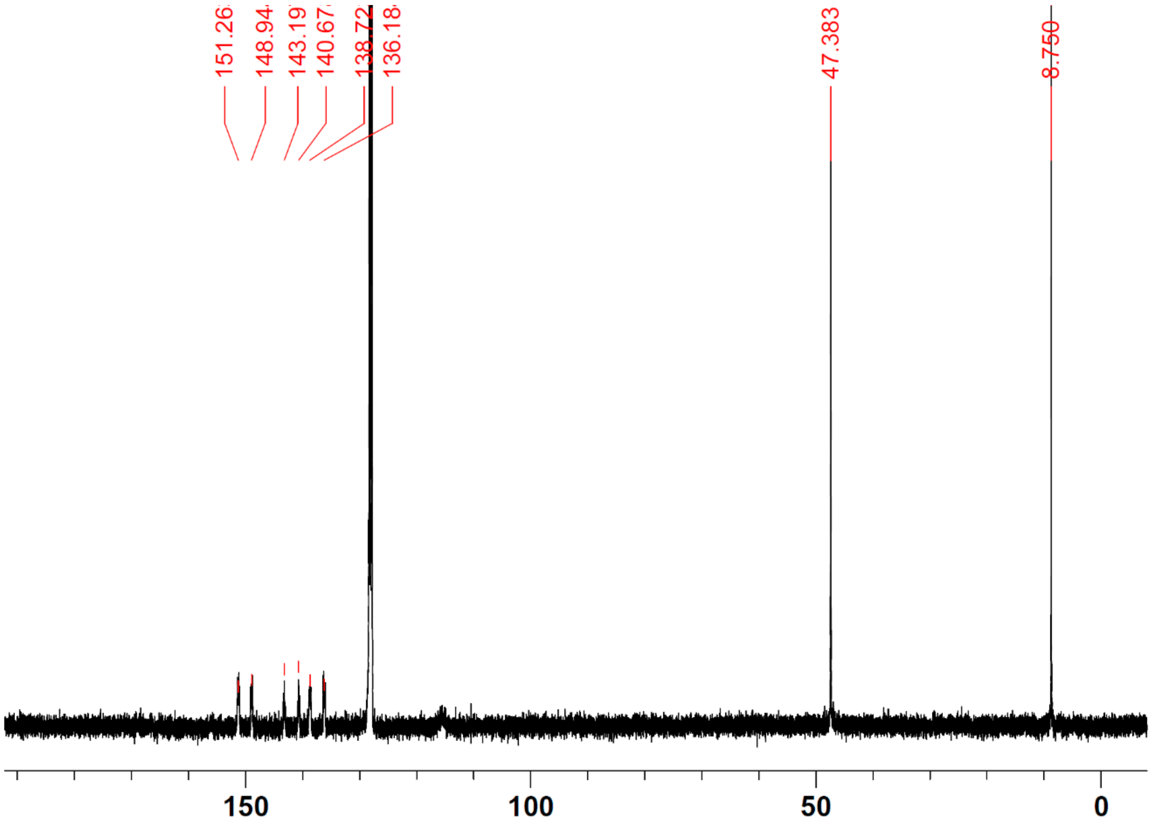

Figure S2. ^{13}C-NMR spectrum of adduct $Et_3N{\cdot}Al(C_6F_5)_3$.

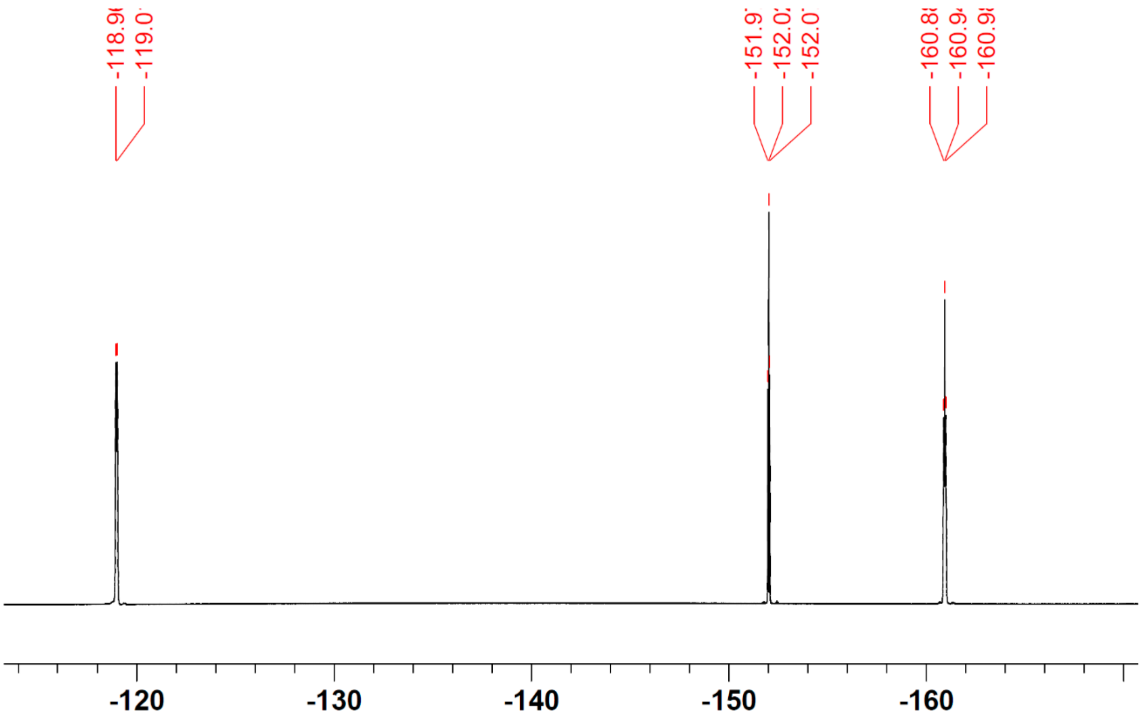

Figure S3. ^{19}F-NMR spectrum of adduct $Et_3N \cdot Al(C_6F_5)_3$.

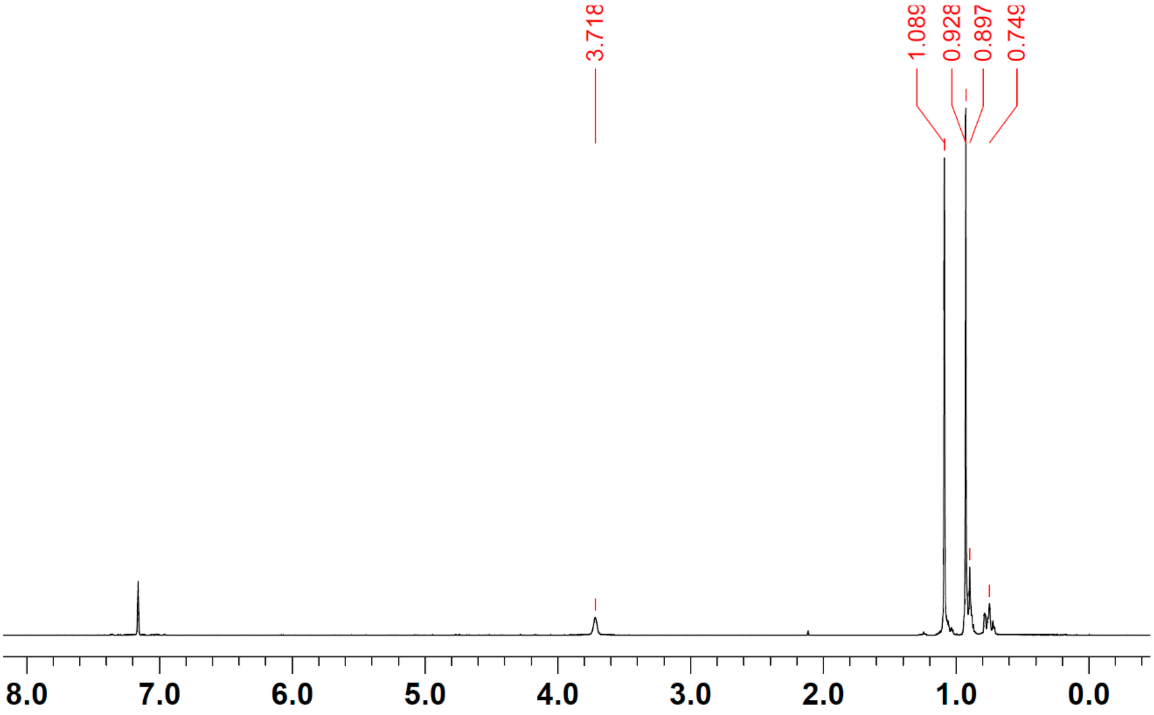

Figure S4. ^{1}H-NMR spectrum of adduct $TMP \cdot Al(C_6F_5)_3$.

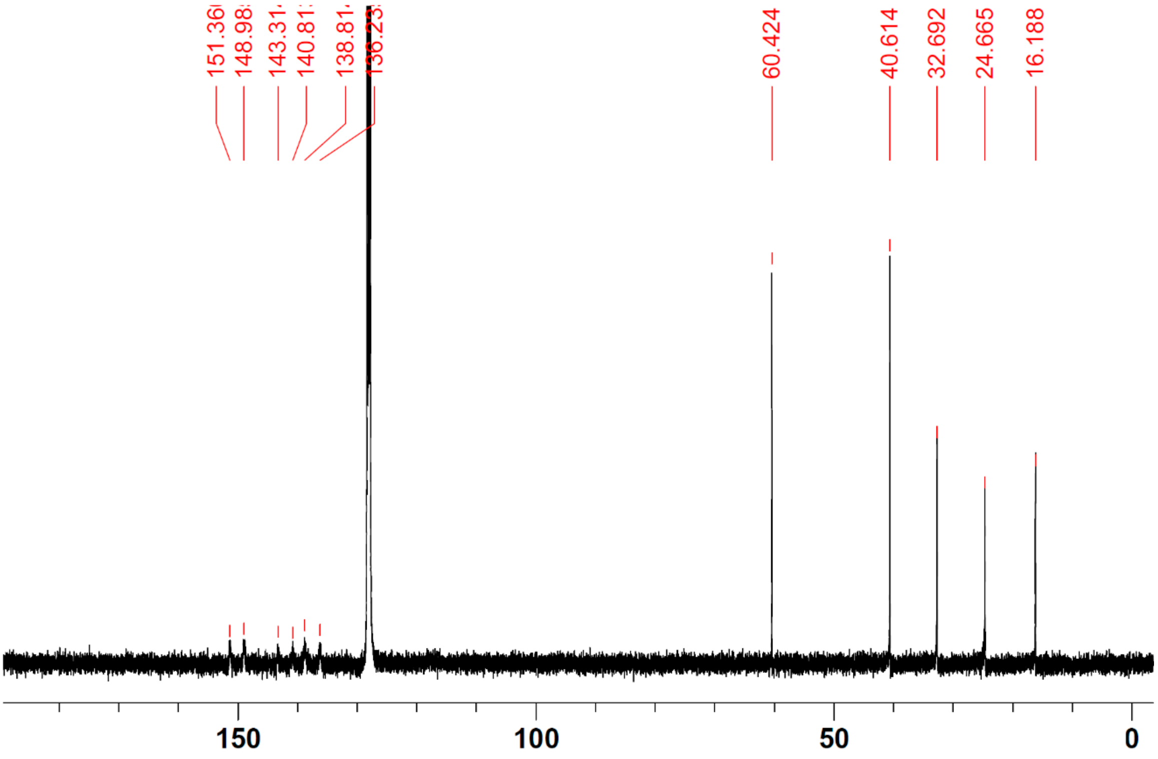

Figure S5. ^{13}C-NMR spectrum of adduct TMP·Al(C_6F_5)$_3$.

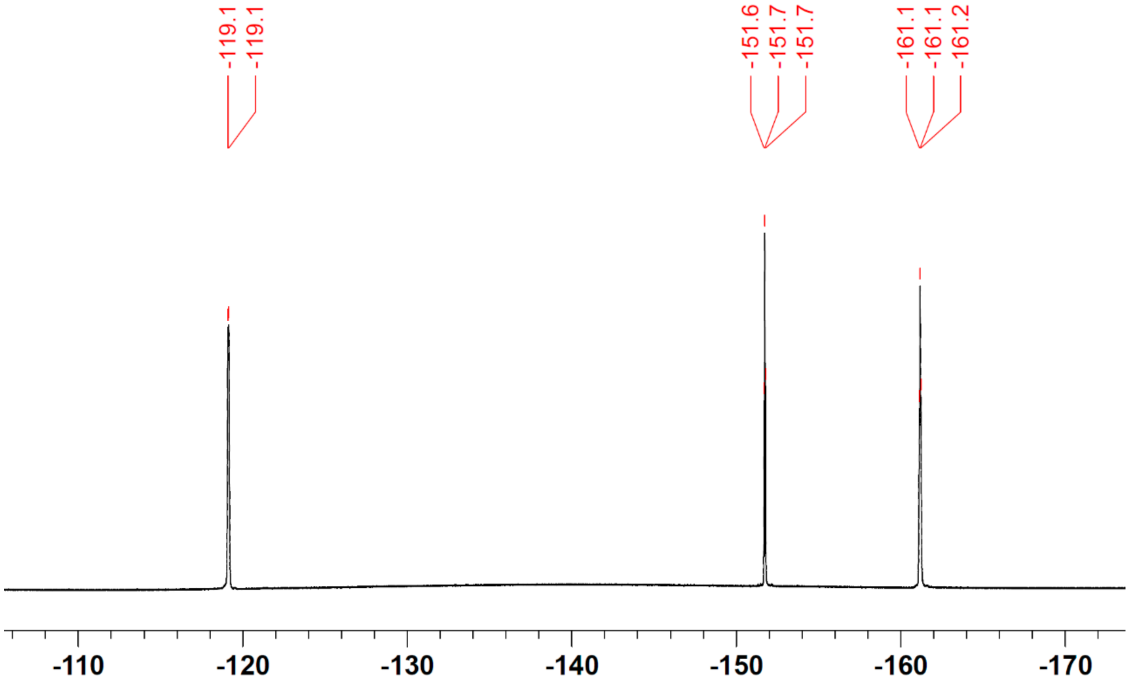

Figure S6. ^{19}F-NMR spectrum of adduct TMP·Al(C_6F_5)$_3$.

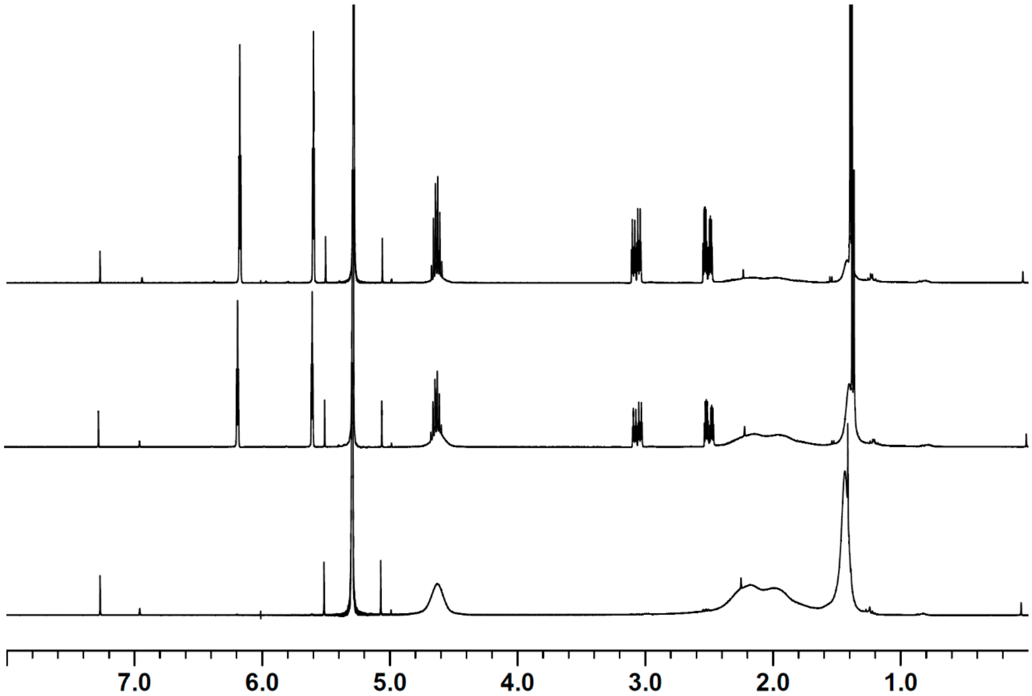

Figure S7. ^{1}H-NMR spectra ($CDCl_3$) of polymerization of γMMBL by $Et_3N/B(C_6F_5)_3$ with a ratio of 800:2:1 extracted at 10, 30, 60 min.

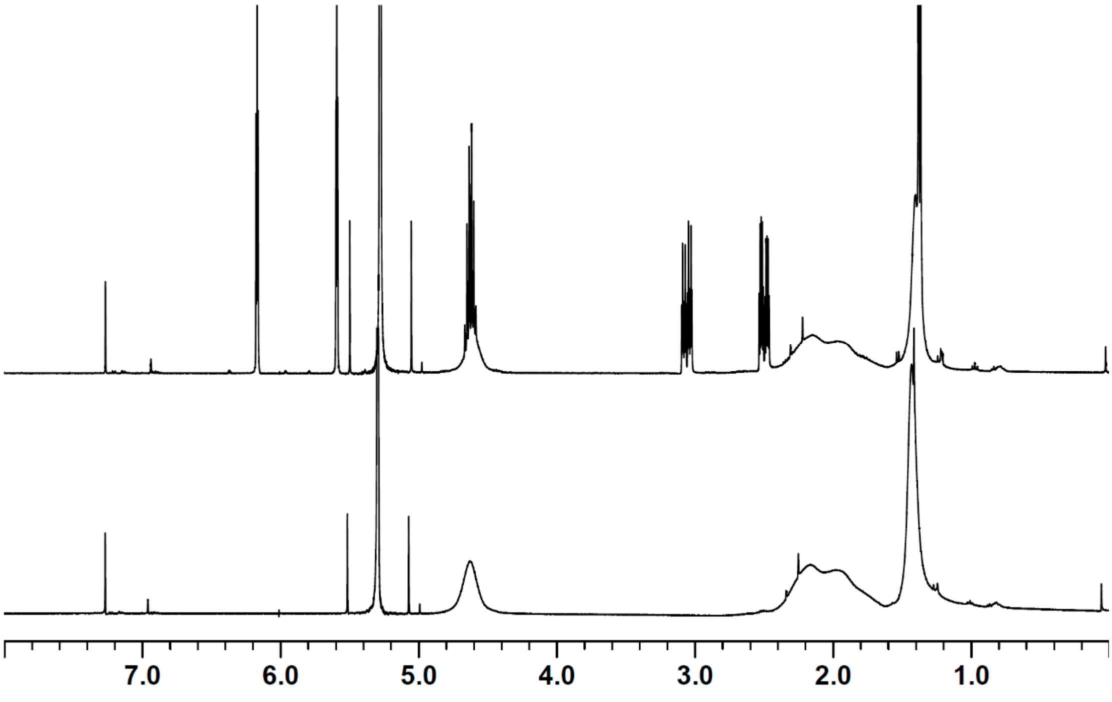

Figure S8. ^{1}H-NMR spectra ($CDCl_3$) of polymerization of γMMBL by $Et_3N/Al(C_6F_5)_3$ with a ratio of 800:2:1 extracted at 10, 30 min.

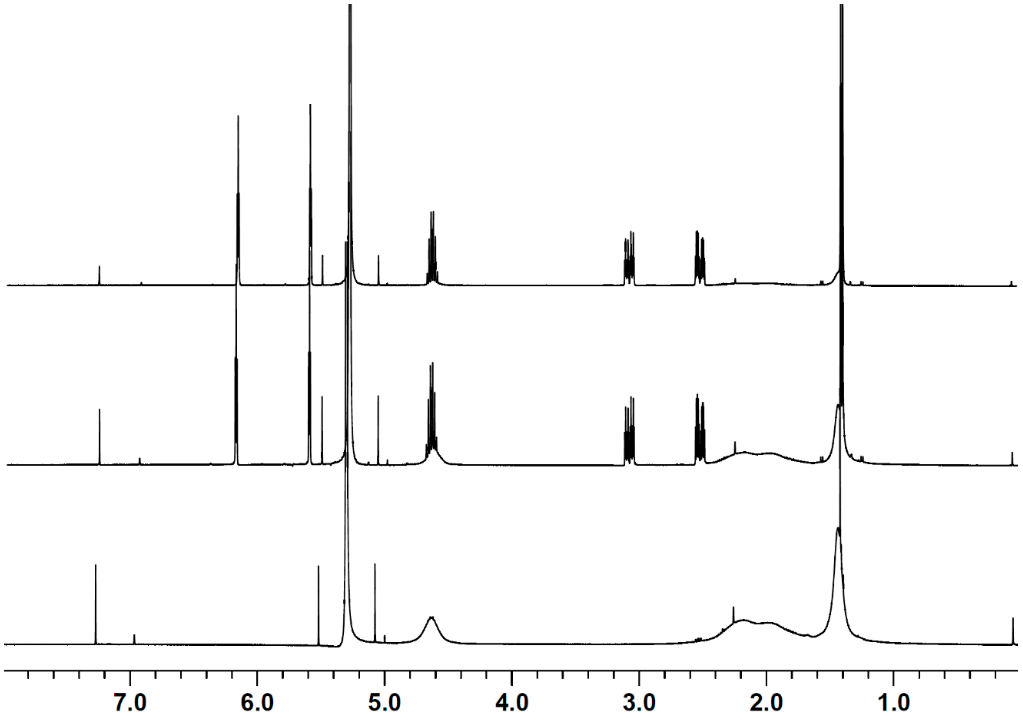

Figure S9. ^{1}H-NMR spectra ($CDCl_3$) of polymerization of $_{\gamma}$MMBL by TMP/$B(C_6F_5)_3$ with a ratio of 1600:2:1 extracted at 1, 2, 3 min.

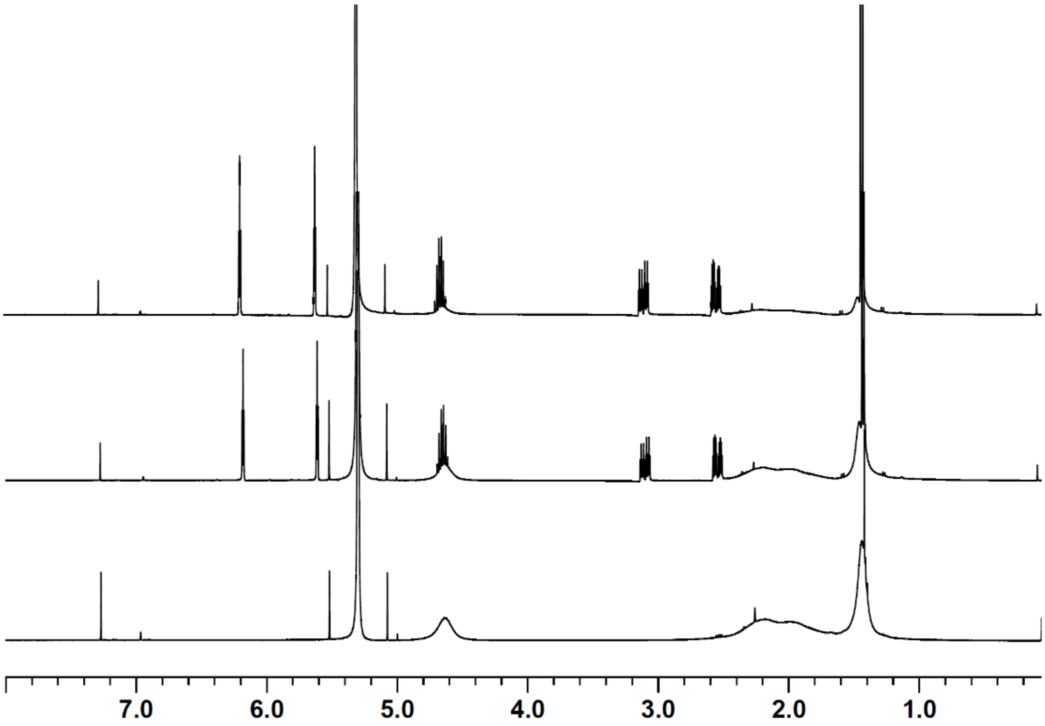

Figure S10. ^{1}H-NMR spectra ($CDCl_3$) of polymerization of $_{\gamma}$MMBL by TMP/$Al(C_6F_5)_3$ with a ratio of 1600:2:1 extracted at 1, 2, 3 min.

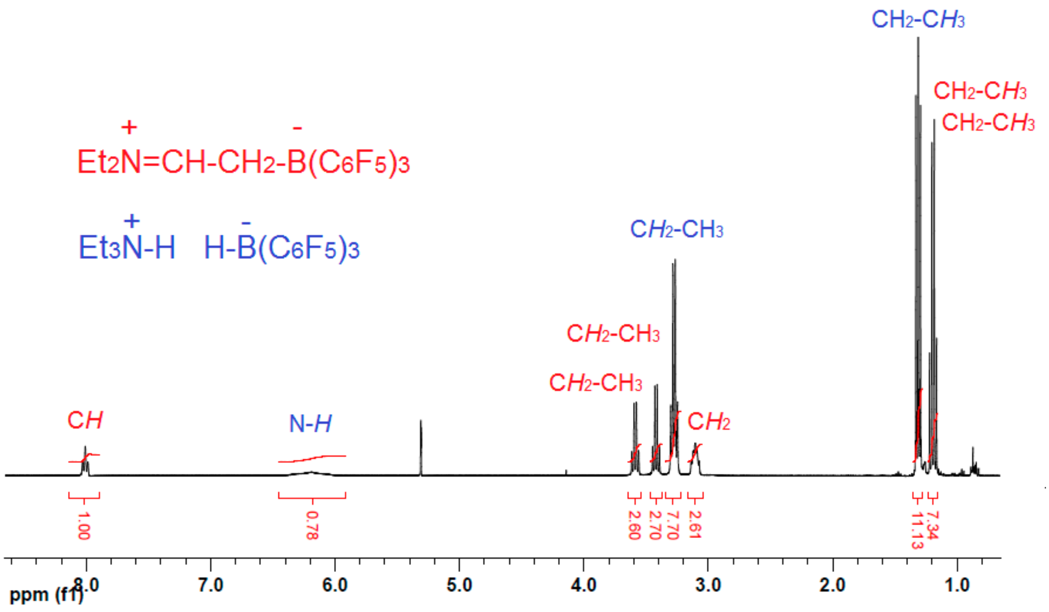

Figure S11. ^{1}H-NMR spectrum in CD_2Cl_2 of a stoichiometric reaction between of Et_3N and $B(C_6F_5)_3$.

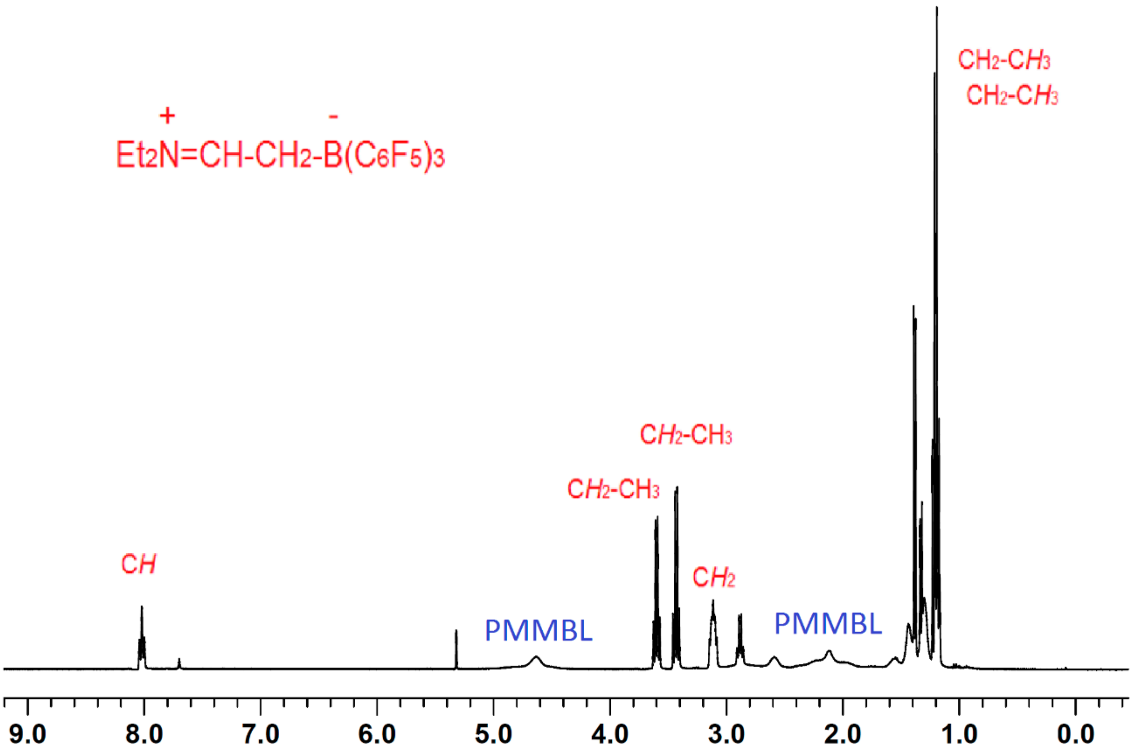

Figure S12. ^{1}H-NMR spectrum in CD_2Cl_2 of a stoichiometric reaction between of Et_3N, $B(C_6F_5)_3$ and $_{\gamma}$MMBL.

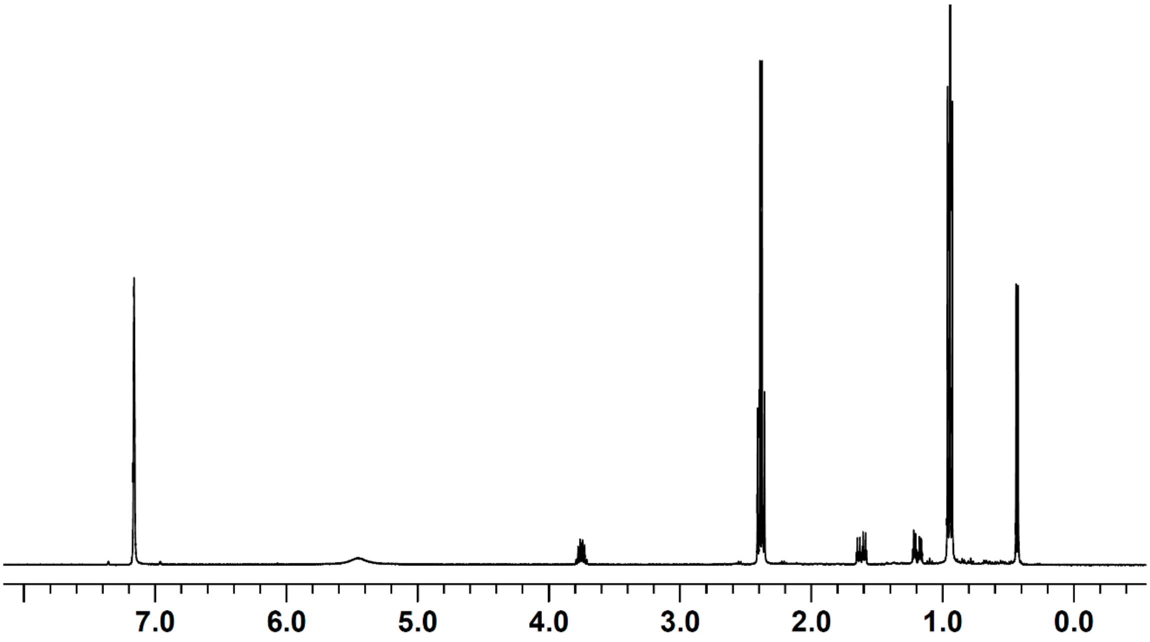

Figure S13. ^{1}H-NMR spectrum in C_6D_6 of a stoichiometric reaction between of Et_3N, $Al(C_6F_5)_3$ and $_{\gamma}$MMBL.

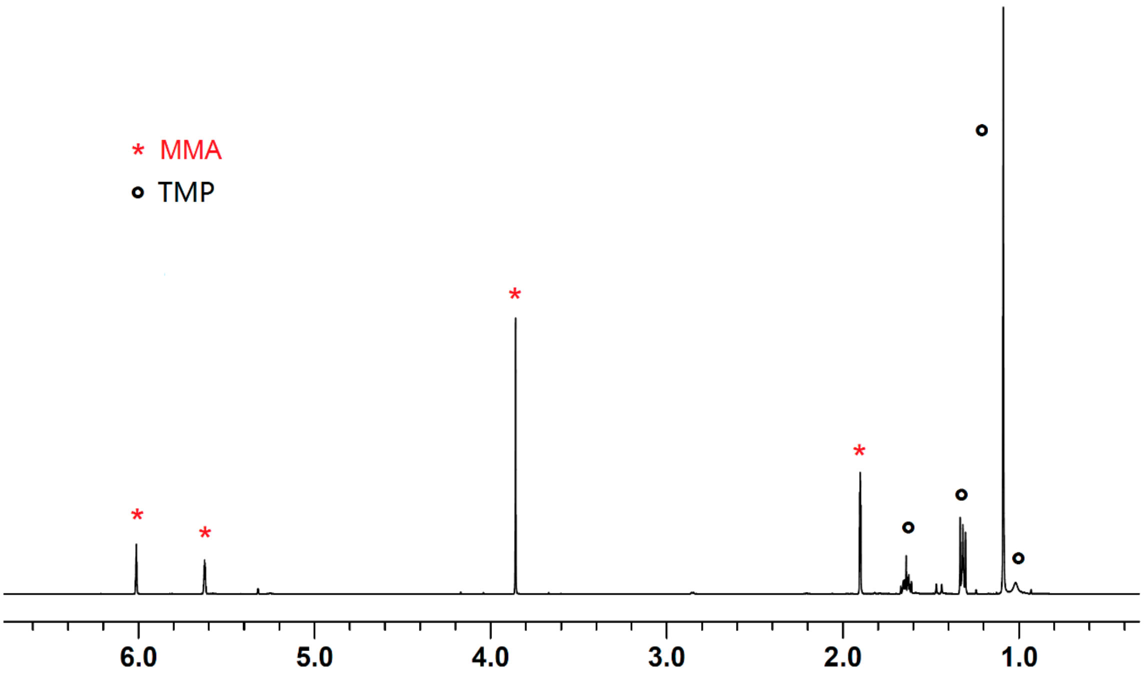

Figure S14. ^{1}H-NMR spectrum in CD_2Cl_2 of a stoichiometric mixture of Et_3N, $B(C_6F_5)_3$ and MMA.
